# AI-powered diagnosis of ovarian conditions: insights from a newly introduced ultrasound dataset

**DOI:** 10.3389/fphys.2025.1520898

**Published:** 2025-07-08

**Authors:** Mahdi-Reza Borna, Hanan Saadat, Mohammad Mehdi Sepehri, Hossein Torkashvand, Leila Torkashvand, Shamim Pilehvari

**Affiliations:** ^1^ Faculty of Industrial Engineering and Systems, Tarbiat Modares University, Tehran, Iran; ^2^ Center of Excellence in Healthcare Systems Engineering, Tarbiat Modares University, Tehran, Iran; ^3^ Fertility and Infertility Research Center, Hamadan University of Medical Sciences, Hamadan, Iran

**Keywords:** ovarian disease diagnosis, transfer learning, CNN, ultrasound imaging, AI-driven diagnostics, multiclass classification

## Abstract

**Introduction:**

Ovarian diseases, including Polycystic Ovary Syndrome (PCO) and Dominant Follicle irregularities, present significant diagnostic challenges in clinical practice. Traditional diagnostic methods, reliant on subjective ultrasound interpretation, often lead to variability in accuracy. Recent advancements in artificial intelligence (AI) and transfer learning offer promising opportunities to improve diagnostic consistency and accuracy in ovarian disease detection.

**Methods:**

We introduced a new, publicly available dataset of ultrasound images representing three ovarian conditions: Normal, PCO, and Dominant Follicle. Using transfer learning, we applied four CNN models—AlexNet, DenseNet121, ResNet18, and ResNet34—to evaluate their performance in multiclass classification of these conditions. The models were assessed using macro and micro metrics, including accuracy, F1 score, precision, and recall, to determine their effectiveness in classifying ovarian conditions.

**Results:**

The results showed that ResNet18 demonstrated the highest performance across all metrics, particularly excelling in the classification of Normal and PCOS conditions. ResNet18 achieved the best performance, with an accuracy of 76.2% and a macro F1-score of 78.2%, demonstrating its effectiveness in distinguishing ovarian conditions. AlexNet also delivered strong results, achieving near-perfect precision in PCOS classification. However, DenseNet121 showed less competitive performance in classifying Dominant Follicle, although it still benefited from transfer learning. The overall results suggest that transfer learning enhances the classification accuracy of CNN models in ovarian disease diagnosis.

**Discussion:**

The application of transfer learning in this study significantly improved the performance of CNN models, especially for Normal and PCOS classifications. The introduction of a publicly available dataset serves as an important contribution to the field, facilitating further research in AI-driven diagnostics. These findings highlight the potential of AI to revolutionize ovarian disease diagnosis by providing more reliable and accurate results, reinforcing the importance of AI in early detection and diagnosis.

**Conclusion:**

This study demonstrates the significant potential of CNN models, enhanced by transfer learning, in improving the diagnostic accuracy of ovarian conditions. The publicly available dataset introduced here will serve as a valuable resource for future research, advancing AI-based medical diagnosis. Further work on refining model architectures and applying these methods in clinical practice is necessary to ensure their reliability and broader applicability.

## 1 Introduction

The accurate diagnosis of ovarian conditions is critical for timely and effective medical interventions, particularly when distinguishing between normal ovarian states and pathologies such as dominant follicles and polycystic ovary syndrome (PCO). Ovarian health plays a central role in female reproductive systems, and disorders such as PCOS can lead to significant complications, including infertility, metabolic disorders, and an increased risk of endometrial cancer. The differentiation between normal ovarian conditions and these pathologies is essential, yet challenging due to the variability in ovarian morphology, especially in ultrasound imaging.

Ultrasonography is the most commonly used imaging modality for evaluating ovarian health due to its non-invasive nature, accessibility, and cost-effectiveness. However, manual interpretation of these images can be time-consuming and subject to human error, particularly in distinguishing between normal and pathological ovaries. Subtle differences between conditions like Dominant Follicle and PCOS require a high level of expertise and consistency, which is often difficult to achieve in routine clinical practice. As a result, there is an increasing interest in developing automated methods for ovarian classification.

Recent advancements in deep learning have shown immense potential in medical image analysis. Convolutional neural networks (CNNs), in particular, have demonstrated their ability to outperform traditional methods in a variety of classification tasks. Deep learning models can automatically extract relevant features from imaging data, providing more accurate and consistent classifications. The application of such models in ovarian classification tasks has the potential to significantly improve diagnostic accuracy while reducing the burden on radiologists.

In this study, we aim to leverage CNN methods to classify ovarian conditions into three distinct categories: Normal, Dominant Follicle, and PCO. These categories represent important clinical states that require different management approaches. Normal ovaries reflect healthy reproductive function, whereas dominant follicles are associated with ovulatory cycles. On the other hand, PCOS is characterized by the presence of multiple small cysts and is a key feature of polycystic ovary syndrome, a condition that affects a significant percentage of women of reproductive age.

Given the complexity of distinguishing between these classes, we have employed a range of CNN models, including AlexNet, DenseNet121, ResNet18, and ResNet34, to perform the classification task. Each of these models offers unique strengths in feature extraction and learning capacity, making them suitable for different aspects of this problem. Our dataset consists of ultrasound images with labeled classes, and we have trained these models to identify the subtle features that distinguish between Normal, Dominant Follicle, and PCO.

This paper presents the results of our CNN-based approach, comparing the performance of different models and evaluating their effectiveness in classifying ovarian conditions. By doing so, we hope to provide a valuable tool that can assist clinicians in diagnosing ovarian health more accurately and efficiently, ultimately improving patient outcomes.

## 2 Related works

Several studies have explored the use of deep learning (DL) models to improve ovarian condition diagnosis, showing promising advancements in both accuracy and applicability (Wang et al., 2023). developed a deep learning model based on U-net++ architecture to differentiate between borderline ovarian tumors (BOT) and epithelial ovarian cancer (EOC) using MR images. The model achieved a higher accuracy (83.7%) than radiologists in distinguishing between the two conditions, proving the potential of DL methods to outperform traditional medical assessments.

(Fan et al., 2023) focused on diagnosing ovarian cysts through ultrasound imaging, a prevalent method due to its non-invasiveness. The authors introduced Ocys-Net, a lightweight network incorporating advanced feature extraction techniques, achieving a high classification accuracy of 95.93%. The study demonstrates the effectiveness of DL in reducing the workload of physicians by enabling rapid and accurate ovarian cyst classification.

In a comprehensive effort (Cho et al., 2024), developed a deep learning model to classify benign and malignant ovarian cysts by integrating ultrasound images with clinical data such as patient age and tumor markers (CA-125 levels). By using ResNet50 and DenseNet121 architectures, the model significantly improved diagnostic accuracy, achieving AUCs of up to 0.96 when clinical and imaging data were combined. The study highlights the value of combining multimodal data for improved ovarian cancer detection.

A systematic review analyzing 96 DL-driven studies on ovarian cancer diagnostics revealed several important trends (Hira et al., 2023). Most studies (71%) focused on detection and diagnosis, with limited emphasis on prediction or prevention. The review also found a lack of model validation using diverse datasets and minimal consideration of AI assurance (AIA). The results highlight the need for more robust and generalizable DL models in ovarian cancer research.

In another study, a convolutional autoencoder (CNN-CAE) was employed to classify ovarian tumors using 1,613 ultrasound images (Jung et al., 2022). The CNN-CAE model effectively removed extraneous elements from images and achieved impressive results, including 97.2% accuracy in classifying ovarian tumors and distinguishing malignant conditions with an AUC of 0.94. The study demonstrates the robustness of CNN models in ovarian tumor classification.

A retrospective, multicenter study collected pelvic ultrasound images from ten hospitals to develop a deep convolutional neural network (DCNN) for ovarian cancer diagnosis (Gao et al., 2022). The model showed improved diagnostic accuracy compared to radiologists, with an AUC of 0.911 in internal datasets and even higher results when the DCNN-assisted diagnosis was employed. This demonstrates the capability of DL models to augment medical expertise, improving accuracy in diagnosing ovarian cancer.

In a similar study (Jan et al., 2023), integrated CT image-based radiomics and DL features to classify benign and malignant ovarian tumors. The best-performing ensemble model combined these features and outperformed junior radiologists, increasing diagnostic accuracy from 66% to 82%. This approach highlights the potential for DL-assisted radiomics to enhance the performance of less-experienced practitioners.

Ziyambe et al. (2023) leveraged CNN models trained on histopathological images to tackle the challenge of diagnosing ovarian cancer, achieving a high accuracy of 94%. The model significantly reduced inter-observer variability and extended analysis times associated with traditional diagnostics, offering a faster, more reliable alternative for predicting ovarian cancer progression.

Expanding the application of DL, a pioneering approach employed transfer learning techniques to detect ovarian cancer and related conditions using histopathological images (Kumar et al., 2024). The study utilized various CNN architectures, including AlexNet2 and EfficientNet, achieving an impressive accuracy of 99.74%, showcasing the potential of advanced segmentation techniques to refine diagnostic precision.

A fuzzy rule-based CNN model was introduced in another study to automate the detection of ovarian cysts using ultrasound images (Ravishankar et al., 2023). This system (OCD-FCNN) achieved an accuracy of 98.37% on benchmark datasets, providing an effective tool for early ovarian cyst detection, essential for timely intervention.

In another study (Chen et al., 2022), developed deep learning algorithms for the automated classification of benign and malignant ovarian tumors using multimodal ultrasound (US) images. Two fusion strategies, feature fusion (DLfeature) and decision fusion (DLdecision), were employed to compare their performance with the Ovarian-Adnexal Reporting and Data System (O-RADS) and expert assessments. DLfeature achieved an AUC of 0.93, which was comparable to the AUC of O-RADS and expert assessment. Both DL models, alongside O-RADS and expert evaluations, reached sensitivities of over 90% for malignancy detection. This study underscores the potential of DL algorithms to match or surpass traditional diagnostic methods in ovarian tumor classification using US imaging.

In another study, deep learning algorithms were developed using multimodal ultrasound (US) images to classify benign and malignant ovarian tumors (Wang et al., 2024). This multimodal approach outperformed single- and dual-modality models, achieving an accuracy of 93.80%, highlighting the value of combining US images with clinical data in ovarian tumor classification.

## 3 Methods

### 3.1 Dataset

The dataset used in this study was collected from Fatemieh Hospital of Hamedan University of Medical Science during the years 2023 and 2024. It consists of **ultrasound images**, each with a resolution of 1,024 × 1,024 pixels, capturing various ovarian conditions. The dataset is organized into three distinct classes: Normal, Dominant Follicle, and Polycystic Ovary Syndrome (PCO). These classes represent crucial clinical states requiring different management approaches, with Normal indicating healthy ovarian function, Dominant Follicle associated with ovulatory cycles, and PCOS characterized by the presence of multiple small cysts, a key feature of polycystic ovary syndrome.

To provide further context, the dataset consists of 301 transvaginal ultrasound images, each corresponding to a unique patient, resulting in a total of 301 women aged between 20 and 45 years. All ultrasound scans were performed using a Philips Affiniti 50 system (Philips, Netherlands). To ensure consistency and clinical accuracy, all images were captured by a single board-certified obstetrician-gynecologist with fellowship training in infertility.

The dataset includes a total of 301 labeled ultrasound images, as shown in Table 1, with 41 images classified as Normal, 144 as Dominant Follicle, and 116 as PCO. The labeling was performed by experienced radiologists, ensuring high-quality ground truth for model training and evaluation.

**TABLE 1:** Distribution of ultrasound images in the dataset across three classes: Normal, Dominant Follicle, and Polycystic Ovary Syndrome (PCO).

Class	Number of images
Normal	41
Dominant Follicle	144
PCO	116
**Total**	301

**FIGURE 1:**
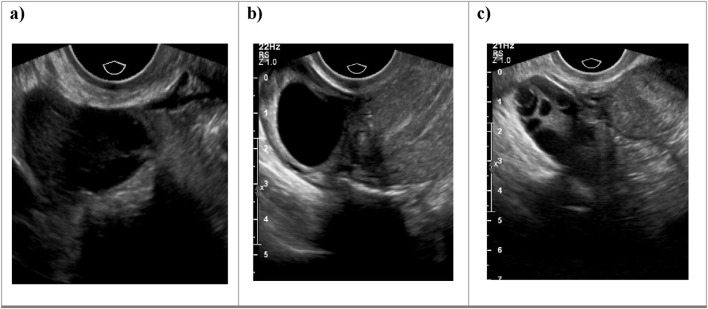
Sample ultrasound images from the dataset, representing the three classes: **(a)** Normal, **(b)** Dominant Follicle, and **(c)** Polycystic Ovary Syndrome (PCO). These examples highlight the visual differences between the classes used for muticlass classification.

Additionally, Figure 1 below presents a sample of ultrasound images from each of the three classes, demonstrating the visual differences between the conditions and the challenges involved in classification.

### 3.2 Data preprocessing

Before training the models, all ultrasound images underwent a standardized preprocessing pipeline to ensure compatibility with the input requirements of the pre-trained CNN architectures. Each grayscale image was first resized to a resolution of 256 × 256 pixels to match the expected input dimensions of the networks. Since the pre-trained models on ImageNet expect three-channel (RGB) input, the single-channel ultrasound images were duplicated across the three channels.

Normalization was applied using the mean and standard deviation values of the ImageNet dataset to align the input distributions with those of the original pretraining environment. This step helps stabilize training and improves convergence. Specifically, pixel values were normalized using the following parameters: mean = [0.485, 0.456, 0.406] and standard deviation = [0.229, 0.224, 0.225].

To improve model generalization and mitigate overfitting, we applied data augmentation techniques during training. These included random horizontal flipping and random brightness adjustments. No additional filtering or denoising techniques were applied, as the raw ultrasound images were deemed sufficiently clean by the expert radiologists who curated the dataset.

### 3.3 Convolutional neural networks models

In this study, we aimed to address a multi-class classification problem, where the goal was to categorize ovarian ultrasound images into one of three distinct classes: Normal, Dominant Follicle, or Polycystic Ovary Syndrome (PCO). To achieve this, we utilized four well-known CNN architectures—AlexNet, DenseNet121, ResNet18, and ResNet34—each combined with transfer learning. These models were selected for their proven effectiveness in image classification tasks and their adaptability to medical imaging applications with limited data.

#### 3.3.1 AlexNet

**AlexNet** was one of the pioneering architectures in deep learning, winning the ImageNet competition in 2012. It consists of five convolutional layers, followed by three fully connected layers, and employs the ReLU activation function to introduce non-linearity. The model’s success was partly due to its use of Rectified Linear Units (ReLU) instead of traditional activation functions like Sigmoid or Tanh, enabling faster training. Additionally, AlexNet uses max-pooling to reduce the spatial dimensions of feature maps, making it computationally efficient. Dropout is applied in the fully connected layers to prevent overfitting, which was a novel approach at the time.

AlexNet is known for its ability to handle large-scale datasets and is well-suited for image classification tasks. In your case, AlexNet can be beneficial due to its relatively simple structure and ability to capture spatial hierarchies within ovarian images. The model’s convolutional layers can extract essential features from the ovarian images, such as textures or shapes indicative of abnormalities, and the fully connected layers can perform the classification based on these features. Despite being an older model, AlexNet remains a solid choice for baseline performance (Krizhevsky et al., 2012).

#### 3.3.2 DenseNet 121

**DenseNet 121** belongs to the family of densely connected convolutional networks (DenseNets). One of the key innovations of DenseNet is its dense connectivity pattern, where each layer receives input from all preceding layers. This structure helps alleviate the vanishing gradient problem and promotes feature reuse, as the network can access features from previous layers without the need for duplication. DenseNet 121, specifically, is a lighter variant of the original DenseNet, making it computationally efficient while maintaining high accuracy.

DenseNet 121 is well-suited for medical image analysis tasks because its dense connections allow for better feature propagation, which is critical for identifying subtle variations in ovarian tissue morphology. In ovarian tumor classification, for instance, DenseNet 121s ability to access a richer variety of features could help the model discern between benign and malignant tumors more effectively. Additionally, its use of fewer parameters makes it less prone to overfitting, which is essential when dealing with medical datasets of limited size (Huang et al., 2018).

#### 3.3.3 ResNet18

**ResNet18** is one of the simpler architectures within the ResNet (Residual Networks) family, which introduced residual connections to mitigate the vanishing gradient problem in very deep networks. The core idea behind ResNet is the use of identity shortcuts, allowing the model to bypass certain layers, thus enabling it to train deeper networks without performance degradation. ResNet18 consists of 18 layers, primarily built from convolutional blocks, and utilizes these residual connections to ease the training of deeper networks.

In the context of ovarian classification, ResNet18’s relatively shallow structure compared to deeper ResNets (like ResNet50) is a suitable balance between complexity and performance. Its residual connections help the network learn meaningful features without losing vital information during the training process. ResNet18 can be particularly useful in detecting specific patterns, such as distinguishing normal ovarian tissue from cystic or polycystic regions, due to its efficient feature learning capabilities (He et al., 2015).

#### 3.3.4 ResNet34

**ResNet34** is a deeper version of the ResNet18 architecture, consisting of 34 layers. It shares the same fundamental design principles, using residual connections to allow information to flow more freely across layers and prevent issues such as vanishing gradients. With its additional layers, ResNet34 can learn more complex features compared to ResNet18, making it better suited for tasks that require a more nuanced understanding of the image data.

For ovarian classification, the deeper structure of ResNet34 may prove advantageous when the task involves differentiating between subtle and complex patterns in ovarian tumors. By being able to process more layers, ResNet34 has the potential to capture deeper hierarchical representations of the input data, which can result in higher accuracy for challenging cases, such as distinguishing between closely related ovarian conditions (He et al., 2015).

Table 2 provides a comparative overview of the CNN models used in this study, highlighting key architectural differences, training efficiency, and their potential to handle small datasets in the task of ovarian classification.

**TABLE 2:** Macro performance metrics for all models.

Metric	AlexNet	DenseNet121	ResNet18	ResNet34
Accuracy	0.747	0.659	0.76	0.688
F1 Score	0.758	0.681	0.782	0.719
Precision	0.817	0.724	0.822	0.776
Recall	0.747	0.659	0.76	0.688

#### 3.3.5 Transfer learning

In this study, we utilized transfer learning to increase the strengths of pre-trained models, enabling efficient training on our limited ovarian ultrasound dataset. Transfer learning allows models pre-trained on large-scale datasets like ImageNet to retain useful low-level features, such as edge detection and textures, which are then fine-tuned for the specific task of ovarian classification. By initializing our models—AlexNet, DenseNet121, ResNet18, and ResNet34—we were able to facilitate the learning process and improve the models’ performance on our three-class problem: Normal, Dominant Follicle, and PCO. Fine-tuning the higher layers allowed the models to adapt to domain-specific features while maintaining general visual patterns learned from the large, diverse dataset. This approach not only reduces the risk of overfitting on small datasets but also increases the models’ ability to capture subtle differences between the ovarian conditions.

To implement this approach, we first removed the original fully connected classification layers from each pre-trained model and replaced them with a new structure consisting of a global average pooling layer, two dense layers (with 128 and 64 neurons, respectively), and a final Softmax output layer with three units corresponding to our target classes. Initially, the convolutional base was frozen to preserve generic image features learned from ImageNet. After training the new classifier layers for several epochs, we unfroze the final convolutional block of each architecture for fine-tuning, allowing the models to better adapt to ultrasound image characteristics.

All ultrasound images were resized to 256 × 256 pixels and normalized using ImageNet statistics. We applied data augmentation during training, including random horizontal flipping and brightness adjustments, to enhance generalization. The dataset was randomly split into 70% training, 15% validation, and 15% test sets.

Each model was trained for up to 25 epochs with early stopping (patience = 5), using a batch size of 16. We used the Adam optimizer with a learning rate of 0.0001 and employed cross-entropy loss. Softmax activation was applied to the final output layer for multiclass prediction.

Given the class imbalance in the dataset, class weights were incorporated during training to ensure fair treatment of underrepresented classes. All models were implemented using PyTorch 1.13, and training was conducted on a system equipped with an NVIDIA RTX 3060 GPU.

## 4 Results

The models’ performance in distinguishing between the three ovarian conditions—Normal, Dominant Follicle, and Polycystic Ovary Syndrome (PCO)—was evaluated using several classification metrics, including accuracy, precision, recall, F1 score, and AUC-ROC. These metrics provide a comprehensive overview of each model’s capabilities, focusing on both overall performance and the balance between sensitivity and specificity.

### 4.1 Classification metrics

The performance of the models in distinguishing between images are as follows.

#### 4.1.1 Accuracy

This metric is defined as the ratio of correctly classified images to the total number of images and gauges the overall correctness of predictions and is defined as the ratio of correctly classified images to the total number of images. This metric is expressed as:

$$Accuracy=\frac{TP+TN}{TP+TN+FP+FN}$$

Where TP is true positives, TN is true negatives, FP is false positive, and FN is false negatives.

#### 4.1.2 Precision

Precision calculates the accuracy of positive predictions and is defined as the ratio of true positives to the sum of true positives and false positives:

$$Precision=\frac{TP}{TP+FP}$$

#### 4.1.3 Recall

Recall, also known as sensitivity or true positive rate, calculates the proportion of actual positives that are correctly identified:

$$Recall=\frac{TP}{TP+FN}$$

#### 4.1.4 F1 score

The F1 score is the harmonic mean of precision and recall, providing a balance between the two metrics:

$$F1=2\times \frac{Precision\times Recall}{Precision+Recall}$$

### 4.2 Classification results

This section evaluates the performance of the four models—AlexNet, DenseNet121, ResNet18, and ResNet34—across three ovarian conditions: Normal, PCO, and Dominant Follicle. The models were assessed using key metrics such as accuracy, F1 score, precision, and recall. The results are presented in terms of both macro-averaged and micro-averaged metrics to offer a comprehensive understanding of each model’s performance across all classes.

#### 4.2.1 Macro analysis of model performance

Table 2 presents the macro-averaged performance metrics, where each class is given equal importance. These metrics reflect the models’ abilities to balance the classification performance across all three ovarian conditions.

ResNet18 achieved the highest performance, with an accuracy of 0.760 and an F1 score of 0.782, making it the most balanced model. AlexNet also performed well, with an accuracy of 0.747 and an F1 score of 0.758. In contrast, DenseNet121 struggled the most, showing the lowest accuracy (0.659) and F1 score (0.681).

ResNet34 provided moderate performance, with an accuracy of 0.688 and an F1 score of 0.719, suggesting it can handle complex data but does not outperform ResNet18.

#### 4.2.2 Micro analysis of model performance

Table 3 provides the micro-averaged metrics, which aggregate the results over all individual instances, without weighting by class. These metrics highlight each model’s overall ability to classify correctly across the entire dataset.

**TABLE 3:** Micro performance metrics for all models.

Metric	AlexNet	DenseNet121	ResNet18	ResNet34
Accuracy	0.749	0.662	0.762	0.69
F1 Score	0.76	0.684	0.784	0.721
Precision	0.82	0.726	0.824	0.778
Recall	0.749	0.662	0.762	0.69

As seen in the macro analysis, ResNet18 maintained the best performance, with an accuracy of 0.762 and an F1 score of 0.784. AlexNet followed closely with an accuracy of 0.749 and an F1 score of 0.760. DenseNet121 again underperformed, showing an accuracy of 0.662 and an F1 score of 0.684.

ResNet34 showed intermediate results, with an accuracy of 0.690 and an F1 score of 0.721.

#### 4.2.3 Class-specific performance

This section presents the class-specific performance of the four models—AlexNet, DenseNet121, ResNet18, and ResNet34—in classifying the three ovarian conditions: Normal, PCO, and Dominant Follicle. Tables 3–5 provide the accuracy, F1 score, precision, and recall for each model and class.

**TABLE 4:** Performance for normal (class 0).

Model	Accuracy	F1 score	Precision	Recall
AlexNet	0.78	0.756	0.886	0.78
DenseNet121	0.703	0.743	0.672	0.83
ResNet18	0.78	0.783	0.8	0.78
ResNet34	0.736	0.769	0.702	0.736

**TABLE 5:** Performance for PCOS (class 1).

Model	Accuracy	F1 score	Precision	Recall
AlexNet	0.956	0.833	1	0.956
DenseNet121	0.945	0.8	0.909	0.714
ResNet18	0.956	0.833	1	0.956
ResNet34	0.956	0.833	1	0.956

Table 4 shows the performance of each model when classifying Normal ovaries. ResNet18 demonstrated the best performance for this class, achieving the highest F1 score of 0.783 and an accuracy of 0.780. AlexNet followed closely with an F1 score of 0.756 and similar accuracy. ResNet34 showed slightly lower performance, with an F1 score of 0.769 and accuracy of 0.736. DenseNet121 struggled the most, with the lowest accuracy of 0.703 and an F1 score of 0.743.

Table 5 presents the performance of the models in classifying PCO. All models performed relatively well for this class. AlexNet and ResNet18 both achieved an F1 score of 0.833 and an accuracy of 0.956, reflecting their ability to precisely identify this condition. ResNet34 also performed well, with similar metrics. DenseNet121 lagged slightly behind, with an F1 score of 0.800 and an accuracy of 0.945.

Table 6 summarizes the performance for Dominant Follicle, which proved to be the most difficult class for all models. ResNet18 achieved the best results, with an F1 score of 0.684 and an accuracy of 0.736. AlexNet showed comparable performance with the same F1 score but a lower precision of 0.565. Both DenseNet121 and ResNet34 struggled more with this class, achieving lower F1 scores of 0.500 and 0.556, respectively.

**TABLE 6:** Performance for dominant follicle (class 2).

Model	Accuracy	F1 score	Precision	Recall
AlexNet	0.736	0.684	0.565	0.736
DenseNet121	0.714	0.5	0.591	0.433
ResNet18	0.736	0.684	0.565	0.736
ResNet34	0.736	0.556	0.625	0.736

The class-specific analysis highlights the strengths and weaknesses of each model. ResNet18 consistently delivered the best overall performance, excelling in the classification of Normal and Dominant Follicle, while maintaining strong precision and recall for PCO. AlexNet also performed well across all classes, especially for PCO, making it a reliable alternative. DenseNet121, however, struggled most with the classification of Dominant Follicle, resulting in lower F1 scores and accuracy. ResNet34 showed moderate performance across all classes, balancing precision and recall but falling short compared to ResNet18. These findings emphasize that deeper models like ResNet18 are better suited for handling the complexity of ovarian condition classification.

To further enhance interpretability, we selected one representative test image from each class—Normal, PCO, and Dominant Follicle—and analyzed the predicted class probabilities output by each model. Alongside each sample image, we also provide its corresponding Grad-CAM saliency map based on the best-performing model (ResNet18), which highlights the image regions that influenced the model’s decision. Table 7 presents these results, showing the image, its saliency visualization, and the predicted probabilities assigned by each model. This multimodal view helps illustrate how confidently and consistently each model responds to different ovarian conditions.

**TABLE 7:** Predicted probabilities for representative images of each class across all models. Values are ordered as Normal, PCO, Dominant Follicle. Grad-CAM maps are from ResNet18.

Sample image	Grad-CAM (ResNet18)	AlexNet	DenseNet121	ResNet18	ResNet34
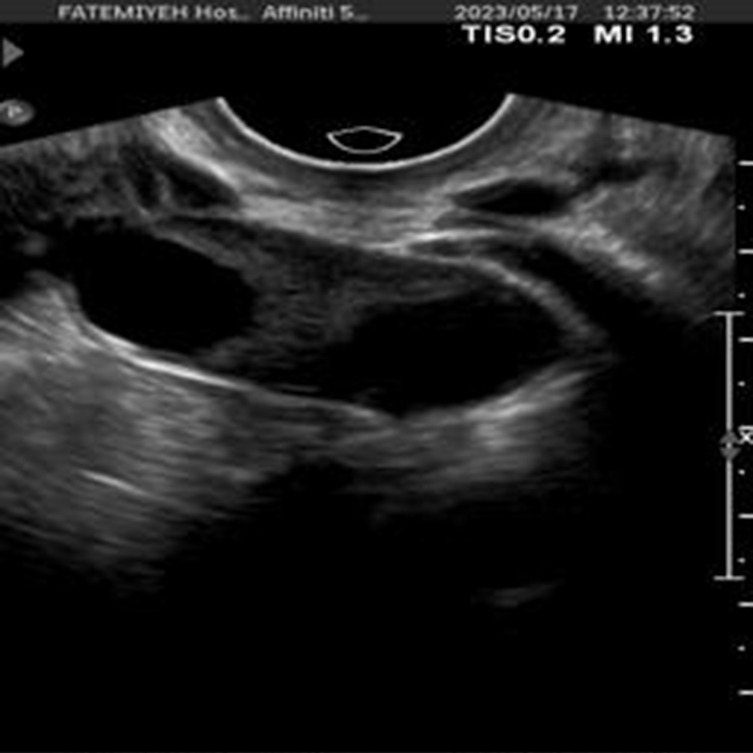	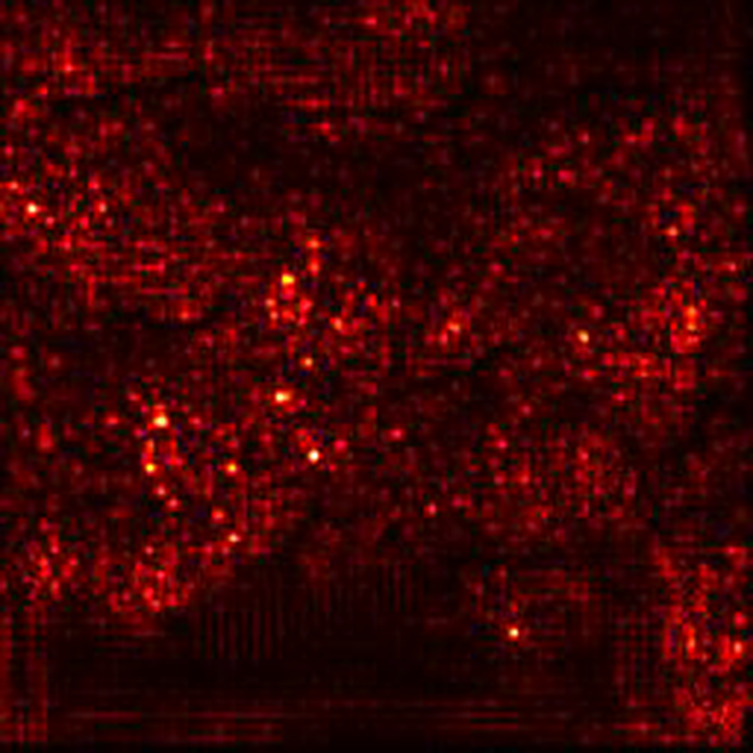	0.88 0.07 0.05	0.85 0.09 0.06	0.91 0.06 0.03	0.86 0.08 0.06
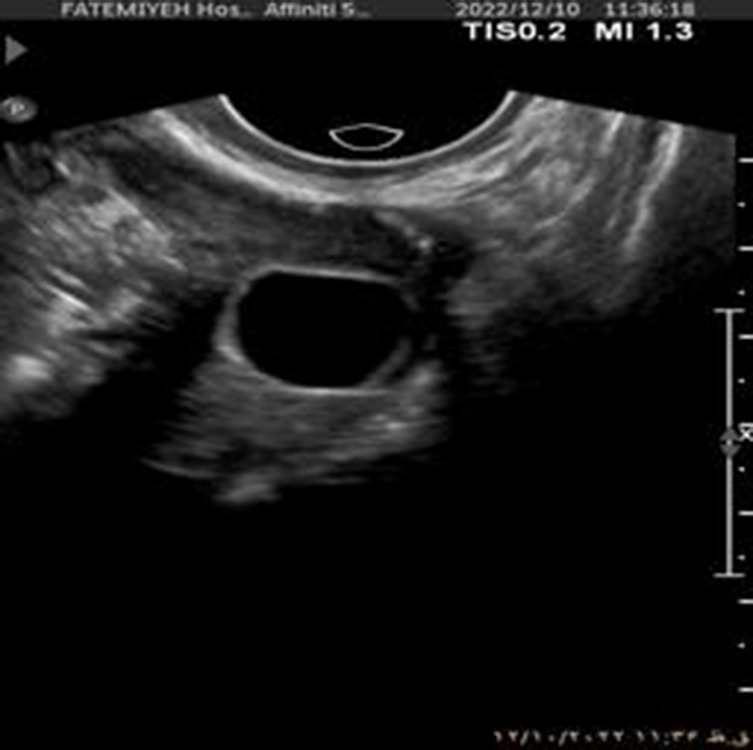	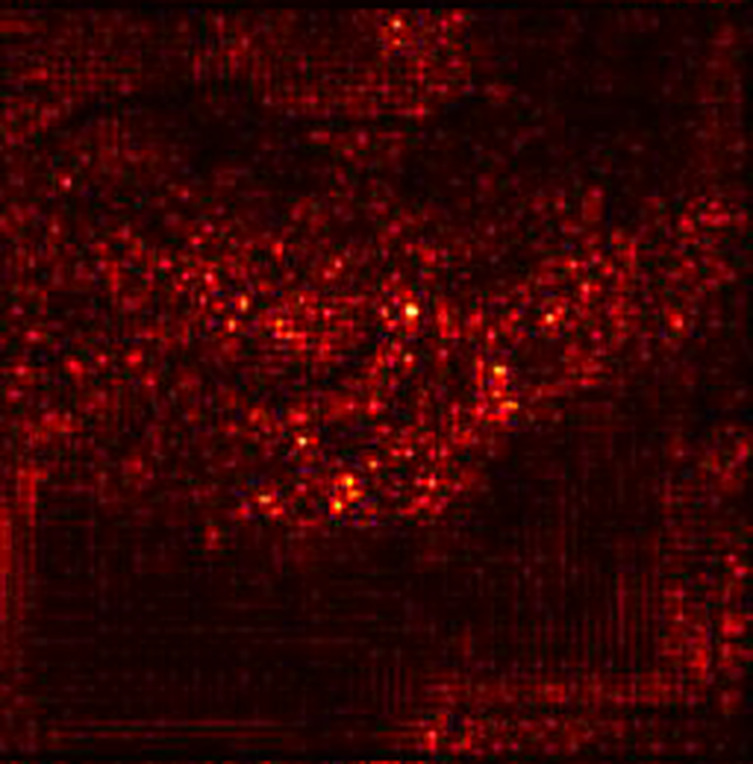	0.01 0.98 0.01	0.12 0.84 0.04	0.02 0.96 0.02	0.06 0.90 0.04
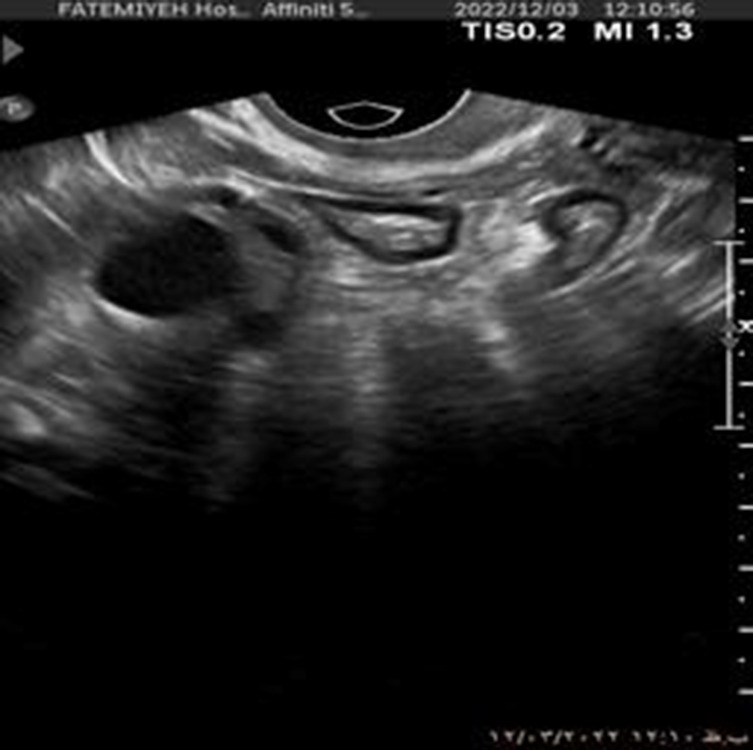	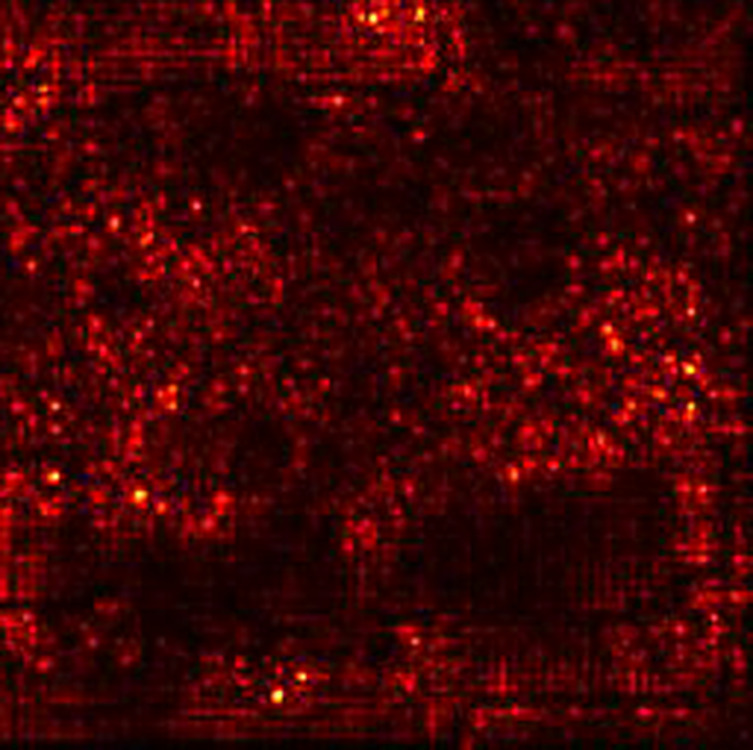	0.26 0.11 0.63	0.34 0.22 0.44	0.20 0.09 0.71	0.30 0.20 0.50

## 5 Discussion

Ovarian diseases, including conditions such as PCOS (Polycystic Ovary), Dominant Follicle irregularities, and other ovarian abnormalities, represent a significant challenge in women’s reproductive health (Ndefo et al., 2013). Early diagnosis is crucial for timely intervention, yet the detection of these conditions through medical imaging, such as ultrasound, can be complex and prone to human error (Serafino et al., 2022). Traditional diagnostic methods often rely on subjective interpretation of ultrasound images, leading to variability in diagnostic accuracy (Pinto et al., 2013). The use of artificial intelligence (AI) and transfer learning in deep learning models has emerged as a promising approach to improving the consistency and accuracy of ovarian disease diagnosis.

In this study, we leveraged transfer learning to evaluate the performance of four CNN models—AlexNet, DenseNet121, ResNet18, and ResNet34—in classifying three ovarian conditions: Normal, PCO, and Dominant Follicle. Our results show that ResNet18, enhanced by transfer learning, consistently outperformed the other models, achieving the highest overall accuracy and F1 scores in both macro and micro metrics. AlexNet, another model fine-tuned using transfer learning, also performed well, particularly in the classification of PCO, where it achieved near-perfect precision. However, DenseNet121 struggled, particularly with the Dominant Follicle class, underscoring the challenges of transfer learning in handling subtle differences in medical images.

The use of transfer learning in medical AI applications raises important ethical considerations (Waisberg et al., 2023). While transfer learning enables AI models to leverage pre-trained knowledge from large-scale datasets, it also introduces concerns regarding data privacy and bias (Jeyaraman et al., 2023; Mennella et al., 2024). Medical imaging data, particularly ultrasound images used in reproductive health, must be handled in compliance with strict privacy regulations to ensure that sensitive patient information is protected. Moreover, models trained on general datasets may not always perform well in specialized medical contexts, potentially leading to biased outcomes if the pre-trained model does not adequately represent all conditions.

In clinical practice, AI models using transfer learning should be integrated as support tools rather than standalone diagnostic systems. While AI can enhance diagnostic precision, particularly in resource-constrained settings, the accountability for diagnoses must remain with healthcare professionals. Transfer learning allows models to be adapted to specific clinical tasks, but clinicians must be aware of the limitations, especially in complex or ambiguous cases, such as Dominant Follicle classification, where the model’s performance was less reliable.

The performance variability observed in this study highlights the need for regulatory frameworks to ensure that AI models, including those based on transfer learning, are robust and reliable before being deployed in clinical settings. Transfer learning, though powerful, must be applied carefully in healthcare, ensuring that the models are sufficiently trained and validated on medical data to avoid discrepancies in patient care.

Although the use of transfer learning improved the overall performance of the models, this study underscores the need for further research in this field. The variability in model performance, particularly for the Dominant Follicle class, suggests that additional training on larger, more diverse medical datasets is needed to enhance the model’s ability to generalize across all ovarian conditions.

One area that warrants future study is the explainability of transfer learning models in medical applications. While transfer learning can accelerate model training and improve performance, it often operates as a “black box,” offering limited insight into the decision-making process. Additionally, although we used standard ImageNet-based normalization in this study to align with common practice in transfer learning, we recognize that using dataset-specific normalization—based on the statistical properties of ultrasound images—could potentially yield more clinically grounded results. Future work should explore both explainable AI (XAI) techniques to improve model interpretability and ultrasound-specific preprocessing strategies to further enhance model robustness and reliability.

Moreover, model robustness remains a key challenge when applying transfer learning in clinical environments. While transfer learning allows models to leverage knowledge from large-scale datasets, these models may still face issues when applied to different imaging modalities or varying clinical settings. Further studies should explore the use of domain adaptation techniques to refine pre-trained models for specific clinical applications, ensuring they perform well in diverse healthcare environments.

To support model interpretability, we incorporated Grad-CAM visualizations using the best-performing architecture (ResNet18). These visual explanations highlight the specific regions of ultrasound images that influenced the model’s predictions, helping clinicians understand the basis of the classification. By providing visual cues linked to diagnostic decisions, such methods can improve trust in AI systems and facilitate their integration into clinical workflows. As shown in Table 7, combining probability outputs with saliency maps offers a more transparent view of model behavior across different ovarian conditions.

The integration of AI and transfer learning in ovarian disease diagnosis holds significant promise for improving diagnostic accuracy and providing faster, more accessible care. By enabling models to transfer knowledge from general datasets to specialized tasks, transfer learning reduces the need for extensive labeled medical data, making it a valuable tool for advancing healthcare AI.

However, to realize the full potential of AI-driven diagnosis, standardized protocols for model development and validation are needed. Collaboration between data scientists, clinicians, and regulatory bodies will be essential to ensure that AI models trained with transfer learning meet rigorous clinical standards. Additionally, the cost-effectiveness and scalability of these AI models must be evaluated, particularly for deployment in resource-limited settings.

## 6 Conclusion

In this study, we introduced a new dataset of ultrasound images representing three ovarian conditions—Normal, PCO, and Dominant Follicle—and made it publicly available for the broader research community. We utilized transfer learning to evaluate the performance of four CNN models: AlexNet, DenseNet121, ResNet18, and ResNet34. Our results demonstrated that ResNet18 consistently outperformed the other models in both macro and micro metrics, particularly excelling in the classification of Normal and PCOS conditions. AlexNet also showed strong performance, especially in PCOS classification, with near-perfect precision. However, the classification of Dominant Follicle proved challenging for DenseNet121, highlighting the limitations of transfer learning in handling subtle variations in medical images.

The introduction of this new, publicly available dataset offers a valuable resource for further research in ovarian condition classification. Our findings suggest that combining deep learning models with transfer learning has significant potential to enhance diagnostic accuracy in clinical settings. However, the variability in model performance across different conditions underscores the need for further refinement, particularly in improving models’ ability to detect more complex conditions like Dominant Follicle.

Future studies should focus on expanding this dataset, exploring new model architectures, and integrating explainable AI (XAI) techniques to provide clinicians with greater transparency into the decision-making process. These advancements will help bridge the gap between AI models and clinical practice, ensuring that AI-assisted diagnosis is both reliable and interpretable.

In conclusion, while this study marks an important step toward improving ovarian condition classification using AI, additional research is needed to refine these models and enhance their applicability in real-world healthcare environments. The publicly available dataset introduced here will contribute to ongoing developments in this critical area of medical research.

## Data Availability

The dataset used and analyzed during the current study is publicly available at https://github.com/HananSaadat/ovarian_ultrasound_dataset. We encourage other researchers to use this dataset to validate our findings and for further investigation.
